# Chicago Medical Society

**Published:** 1882-10

**Authors:** 


### Society Reports.

### ARTICLE VII.

CHICAGO MEDICAL SOCIETY. Stated Meeting, October 2, 1882.

Dr. J. H. Hollister, President, in the Chair.

The President made a few appropriate remarks, welcoming the members to the regular re-opening of the meetings of the society after their summer vacation; and the secretary read the minutes of the last meeting, which were voted approved.

A discussion of rupture of the kidney being in order, Dr. R. G. Bogue remarked that the injury should be looked upon as one of rare occurrence, and most liable to prove fatal. One case had come under his observation, which resulted in death in four or five days. Referring to Dr. Isham's paper on the subject at the last meeting, he commended the doctor's suggestion to establish a fistulous opening for the drainage of the urine, as its extravasation led to gangrene, and brought about the fatal result.

Dr. James H. Etheridge read a paper on removal of both ovaries, published in this issue of the JOURNAL. A lively discussion ensued. Dr. T. D. Fitch was in favor of the operation when indicated.

Dr. E. W. Sawyer had seen Battey's operation performed several times. One patient on whom Dr. Battey operated some years ago, had told him that all sexual desires had been abolished in her by that operation. An earlier appearance of hair on the face, and various signs of premature old age, were also to be seen in such cases.

Dr. Roswell Park, just returned from an extensive trip in Europe, had discussed the subject with the leading gynæcologists of the Continent. Opinions differed. A former assistant of

Lawson Tait had made the remark that as yet, all the operations of oöphorectomy had been performed by young men; and, Dr. Park added, the present instance is not an exception to the rule.

Dr. E. Ingals had witnessed the operation hundreds of times in the case of swine. It was altogether devoid of danger, required no skill, and was invariably successful. It was of the same import as castration in the male, and in either case, the operation was followed by cessation of sexual acts, and anything pertaining thereto.

Dr. Brower's paper on toy pistol tetanus was now read (see another part of the JOURNAL).

In the discussion which ensued, Dr. Belfield said that the only proof of the existence of bacteria in a fluid were: 1. Their reaction to staining agents; 2. Their cultivation in certain other fluids; and 3. Their mode of development or life history. It seemed to him that, in two diseases at least, they played an all-important rôle. He had given the subject much attention, and with the best advantages had pursued its study under the great masters. He had seen bacteria cultivated under the sealed glasses of microscopes, then inoculated into healthy animals, a rapid multiplication of these bacteria had taken place in the blood of these animals, soon resulting in death. The bacillus of splenic fever, and the fungus of active mycosis had ceased to rank with doubtful organisms. Much discredit had been brought on the subject of bacteria by the rash assertions of physicians who, not being microscopists, had derived erroneous views from their untrustworthy observations, and he placed Klebs in that category; but he considered Koch worthy of a good deal of confidence, considering the long space of time he had devoted to his investigations.

Prof. Lester Curtis asserted that there could be no doubt entertained in regard to the bacterial nature of the organisms which he had obtained from the blood of Dr. Brower's patient. He had cultivaled them under sealed glasses for days under the microscope. They rapidly developed into chains of eight or ten joints. These chains generally held the blood corpuscles. The proper precaution had been taken to exclude the presence of bacteria from other sources during the experiment.

Dr. Roswell Park remarked that there was a tendency among some pathologists to class tetanus among the infectious diseases. As to bacteria, some micrococci, and rod bacteria, were generally found in the discharges of healthy wounds, and seemed of no account. In Dr. Brower's case, the treatment most efficacious would rather indicate that tetanus is an infectious disease; yet it was possible that the contagium had escaped even the microscropical observations of the writer, since the bacteria found in that case seemed harmless.

To a query of Dr. Etheridge, Dr. Brower said that he considered toy pistol tetanus the ordinary traumatic form, and he doubted whether tetanus could ever be idiopathic. He looked upon the disease as one primarily involving the end organs of the nervous system, and approved of cutting or stretching the nerves concerned.

---

THE College of Physicians and Surgeons opened their new building with great *eclat* on Tuesday, September 26, 1882. Prof. A. Reeves Jackson delivered the opening address. Prof. S. A. McWilliams and Mayor Harrison made speeches appropriate to the occasion. The class is very large for a new enterprise; one hundred and twenty matriculated.

THE CHICAGO MEDICAL COLLEGE opened its winter session on Tuesday, September 26, 1882. The introductory address was delivered by Prof. M. P. Hatfield. The faculty of the college has been materially strengthened since its last session, and many improvements made in the college building.

THE WOMAN'S MEDICAL COLLEGE held its opening exercises September 25. Prof. E. F. Ingals delivered the opening address to the assembled students, showing them, in well chosen language, the best course to pursue in the study of medicine.

